# Neuromodulation and SCAN holding hands

**DOI:** 10.1093/scan/nsab102

**Published:** 2021-09-04

**Authors:** Paulo S Boggio

**Affiliations:** Social and Cognitive Neuroscience Laboratory, Mackenzie Presbyterian University, Sao Paulo 01241-001, Brazil

**Keywords:** neuromodulation, tDCS, TMS, transcranial stimulation

At Social Cognitive Affective Neuroscience (*SCAN*)’s birth in 2006, Matthew Lieberman, proud dad and Editor-in-Chief, wrote in his editorial that it was not too long ago that the medical and biological sciences had little reason to share a breadcrumb with those in the social sciences. *SCAN* was born like this: in an environment enriched by disciplinary areas that met and joined together in a great interdisciplinary endeavor. While *SCAN* was born and took its first steps, non-invasive brain neuromodulation techniques were resurrected and renewed by the technological advances of the last decades. This special edition is *SCAN*’s encounter with these techniques that, initially more restricted to the arsenal of neurophysiology or clinical neuroscience, have become part of the toolbox of researchers in social and affective neuroscience.

One of the techniques present in the articles that are part of this special issue is transcranial direct current stimulation (tDCS). It is a technique based on the application of low-intensity direct current on the scalp. The use of electricity in medical treatments dates back to ancient Rome. Scribonius Largus used the torpedo fish, which produces electrical discharges, to treat headaches. Could you imagine being treated with fish applications over your head? Interestingly, some cases required more than one fish for treatment to be effective—perhaps already showing one of the basic principles of psychopharmacology, i.e. some effects depend on dose–response–individual relationships. At that time, Seneca was writing, among many other topics, about the importance of friendship, a topic of great importance in current studies in Social and Affective Neuroscience (SAN). Obviously, no one applied an electric fish to study the role of cortical structures in bonding back then, but this is a possible reality these days.

Taking a time warp (from 50 AD to the eighteenth century), but not so much in space (from Rome to Bologna and Pavia), Italy starred in one of the great revolutions in human history: the discovery of the pile. Alessandro Volta departed from Luigi Galvani’s work on the existence of electricity in living beings and developed the voltaic pile. Giovanni Aldini, Galvani’s nephew, applied electrical current in patients with melancholy conditions as well as made public demonstrations on its use in cadavers. The application of brain stimulation in affective disorders already signaled a future encounter between neuromodulation techniques and clinical neuroscience. In addition, people’s imaginations were already beginning to gain new contours, characters and technologies with these extraordinary advances of the time. The result of this can be seen in the literature: Mary Shelley’s classic Frankenstein.

In parallel to these advances in physics and biology, Scottish philosopher David Hume was writing *A Treatise of Human Nature*, introducing something that is another prominent theme in current SAN studies: the role of emotions (passions) in reason. All of this happening at the same time creates a rather unusual fantasy: imagine Giovani Aldini and David Hume investigating together the effects of electromagnetic stimulation of the dorsolateral prefrontal cortex on social decision tasks or the ventrolateral prefrontal cortex on emotional regulation tasks and moral judgment. Impossible at the time, reality nowadays.

This brief history is intended to illustrate the beauty of scientific discoveries and possible encounters between different fields of knowledge. Obviously, these encounters depend on the available technologies, the type of current knowledge and the openness people have to new experiences. Many of these encounters are already possible today. *SCAN* has published articles that show the junction of both different knowledge and different technologies.

**Fig. 1.**
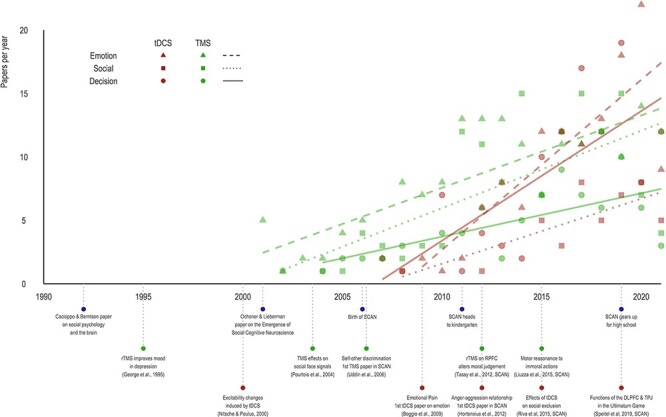
Timeline of social and affective neuroscience and neuromodulation.

Figure 1 provides a simplified timeline showing the emergence and growth of both *SCAN* and non-invasive brain stimulation. I include some milestones that I think are important to give an overview of how areas and techniques have been approaching in recent decades. The 1990s, called the Decade of the Brain, was filled with studies integrating neuroscience with cognitive psychology. But the visionaries John Cacioppo and Gary Berntson in 1994 already signaled the approximation between neurosciences and Social Psychology. At that time, many of us in social neuroscience today were still in undergraduate or PhD programs.

During this period, transcranial magnetic stimulation began to gain prominence. In the 1980s, it gained prominence as an interesting tool for neurophysiologists, and in the 1990s, it began to gain prominence in clinical neuroscience as a possible ally of psychiatry. In 1995, the publication by George *et al.* (1995) marked its debut as a tool for the treatment of depression. It would take a few years for this technique to be used to test hypotheses and propose new advances in our knowledge of SAN.

A few years after this publication, tDCS’ first steps in its recent era were taken. In 2000, the article by Nitsche and Paulus put tDCS on the scene by showing that its application to the scalp modulated cortical excitability. At that time, Felipe Fregni and I were working with Transcranial Magnetic Stimulation (TMS) in studies on depression, but tDCS caught our attention due to its ease of use, high degree of safety and low cost. But a plot twist was decisive in our careers: my father, in the 1970s, had developed tDCS devices for clinical studies in Brazil. What a fascinating discovery when you are at the beginning of your career and without the financial resources to set up your own laboratory. Overnight, we had as many devices as we wanted to conduct new studies. As seen with TMS, new studies from our newly created group as well as others around the world have included tDCS in clinical neuroscience with an emphasis on studies on depression, stroke, Parkinson’s disease and chronic pain.

As we participated in the resurgence of tDCS, Ochsner and Lieberman published in 2001 in *American Psychologist* a pivotal article on the emergence of cognitive and social neuroscience. There, the approach of different areas to understand topics such as emotion regulation, stereotype, emotion interaction and cognition, among others, begins to become evident. From this article to the birth of *SCAN*, 5 years have passed. During this period, TMS was already taking its first steps as a research tool in topics such as processing of emotional faces (Pourtois *et al.*, 2004), and the first volume of *SCAN* already featured an article by Uddin et al. using TMS in a self-other discrimination task. tDCS would take even longer; it was only in 2009 that the first article investigating the effects of tDCS on emotional processing outside the clinical area was published (Boggio *et al.*, 2009), and it was only in 2012 that an article using this technique was published in *SCAN*. At that time, *SCAN* had arrived in kindergarten.

Since then, SAN themes investigated with neuromodulation techniques have expanded rapidly. Figure 1 provides an overview of the number of publications per year on emotional (e.g. emotion regulation), social (e.g. social touch) and decision-making (prosocial decision-making) topics. As the general production has been growing, the presence of neuromodulation techniques in articles published in *SCAN* has also grown. More than 40 articles have been published with TMS and 20 with tDCS. This growth in the area meets the moment when *SCAN* reaches high school. In the ‘*SCAN* Gears up to high-school’ editorial, Editor-in-Chief Matthew Lieberman wrote, ‘Where once I and a few others were referred to the “young Turks” at the start of things, we are now the “greying Turks”’ and also ‘The final change to *SCAN* is that we are going to increase the emphasis on special issues’. Maturity has arrived for everyone, and a special issue on neuromodulation could not fail to happen. May this special edition planned carefully by the ‘greying Turks’ inspire new scientists and advance ever further with the knowledge of social and affective neuroscience.
